# Double knockout of Bax and Bak from kidney proximal tubules reduces unilateral urethral obstruction associated apoptosis and renal interstitial fibrosis

**DOI:** 10.1038/srep44892

**Published:** 2017-03-20

**Authors:** Shuqin Mei, Lin Li, Qingqing Wei, Jielu Hao, Yunchao Su, Changlin Mei, Zheng Dong

**Affiliations:** 1 Kidney Institute and Department of Nephrology, Shanghai Changzheng Hospital, Second Military Medical University, Shanghai, China; 2 Department of Cellular Biology and Anatomy, Medical College of Georgia at Augusta University and Charlie Norwood VA medical Center, Augusta, Georgia, USA; 3 Department of Pharmacology and Toxicology, Medical College of Georgia at Augusta University and Charlie Norwood VA medical Center, Augusta, Georgia, USA; 4 Department of Nephrology, The Second Xiangya Hospital of Central South University, Changsha, China

## Abstract

Interstitial fibrosis, a common pathological feature of chronic kidney diseases, is often associated with apoptosis in renal tissues. To determine the associated apoptotic pathway and its role in renal interstitial fibrosis, we established a mouse model in which Bax and Bak, two critical genes in the intrinsic pathway of apoptosis, were deleted specifically from kidney proximal tubules and used this model to examine renal apoptosis and interstitial fibrosis following unilateral urethral obstruction (UUO). It was shown that double knockout of Bax and Bak from proximal tubules attenuated renal tubular cell apoptosis and suppressed renal interstitial fibrosis in UUO. The results indicate that the intrinsic pathway of apoptosis contributes significantly to the tubular apoptosis and renal interstitial fibrosis in kidney diseases.

Renal interstitial fibrosis is a common pathological feature during the progression of kidney disease to end stage renal failure^1^,^2^. While characterized by interstitial myofibroblast activation and expansion and massive extracellular matrix protein accumulation^3^, renal interstitial fibrosis is often accompanied by renal cell injury and death and inflammation. In fact, both renal cell death and inflammation have been suggested to contribute to the development of renal interstitial fibrosis. However, it is not fully understood whether renal tubular apoptosis is a key factor in interstitial fibrosis and, if it is, what apoptotic pathway is mainly responsible.

There are two major pathways of apoptosis^4^. The extrinsic pathway is initiated by the binding of death receptors, e.g. Fas and TNFαreceptors, by death ligands, leading to the activation of caspase-8 and downstream executioner caspases. In contrast, the intrinsic pathway is centered on mitochondria and thus is frequently referred to as mitochondrial pathway of apoptosis. The intrinsic pathway is characterized by the permeabilization of mitochondrial outer membrane causing the release of apoptogenic factors, especially cytochrome c, which induce the activation of caspase-9 and down-stream caspases^5^,^6^. Bax and Bak are the key proapoptotic Bcl-2 family proteins that are activated in the intrinsic pathway to permeabilize mitochondrial outer membrane for apoptosis^7^. Our previous work has established a critical role for Bax and Bak in renal cell apoptosis in experimental models of acute kidney injury induced by ischemia/reperfusion and cisplatin nephrotoxicity^5^.

In the present study, we have produced renal tubule-specific Bax/Bak double knockout mouse model and tested the effect of Bax/Bak double knockout on tubular apoptosis and renal interstitial fibrosis in the model of unilateral urethral obstruction (UUO).

## Results

**Figure 1:**
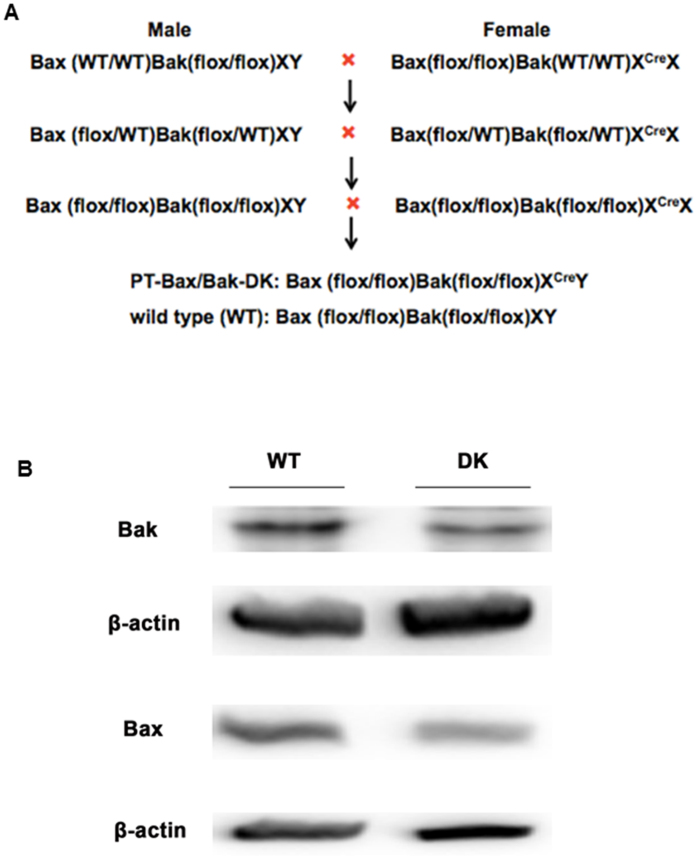
Establishment of proximal tubule-specific Bax and Bak-double knockout (PT-Bax/Bak-DK) mouse model. (**A**) Schematics showing the breeding protocol for generating PT-Bax/Bak-DK mice by crossing Bax(flox/flox)Bak(WT/WT) X^CRE^X mice with Bax(WT/WT)Bak(flox/flox)XY mice. (**B**) Immunoblot analysis of renal cortical tissues to confirm the decreases in Bax and Bak expression in PT-Bax/Bak-DK mice.

In our previously work, we demonstrated that ischemic acute kidney injury was ameliorated in renal proximal tubule Bax knockout mice [PT-Bax(−/−)] mice and global Bak knockout [Bak(−/−)] mice, supporting a critical role of Bax/Bak-mediated mitochondrial pathway of apoptosis^5^. But the role of Bax and Bak in chronic kidney pathology, such as that in UUO, was not entirely clear. To address this, we first generated Bax(flox/flox)Bak(WT/WT) X^CRE^X mice by breeding Bax(flox/flox)Bak(WT/WT)XY and Bax(WT/WT)Bak(WT/WT)X^CRE^X^CRE^(PEPCK-CRE) mice. We then back-crossed Bax(flox/flox)Bak(WT/WT) X^CRE^X mice with Bax(WT/WT)Bak(flox/flox)XY mice for 2 generations to produce proximal tubule-specific Bax/Bak double knockout (PT-Bax/Bak-DK) mice (Fig. 1A). In addition to genotyping results (not shown), Bax and Bak depletion in proximal tubular cells in PT-Bax/Bak-DK mice was confirmed by Western blot analysis (Fig. 1B). Of note, there were residual Bax and Bak signals in the renal cortical tissues in PT-Bax/Bak-DK mice, because the knockout was restricted in proximal tubules and the expression of Bax and Bak in other cell types was not affected.

### Double knockout of tubular Bax and Bak inhibits UUO-induced renal apoptosis

**Figure 2:**
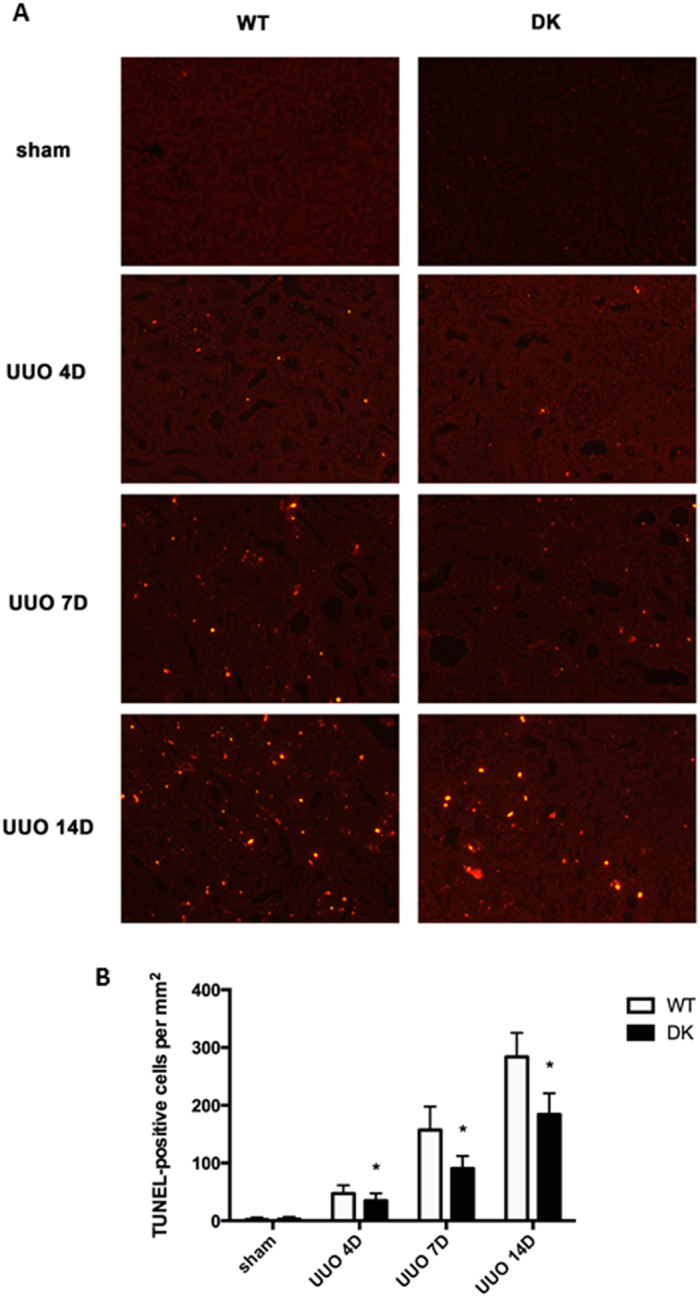
UUO-associated renal tubular apoptosis is alleviated in PT-Bax/Bak-DK mice. PT-Bax/Bak-DK and littermate WT mice (10–12 weeks old) were subjected to UUO or sham surgery, and then sacrificed to harvest the kidneys at 4, 7 and 14 days, respectively. (**A**) Representative images of TUNEL staining. (**B**) Quantification of TUNEL positive cells in kidney tissues. Data were expressed as mean ± SD. *P < 0.05, significant difference between WT and PT-Bax/Bak-DK groups.

UUO is a commonly used model for kidney fibrosis research. The pathological changes in UUO include tubular cell injury and death, phenotypic alterations of renal cells, and interstitial inflammation^8^. By TUNEL staining, we detected less apoptotic cells in PT-Bax/Bak-DK kidney tissues than WT kidney tissues after UUO 4, 7, and 14 days (Fig. 2A and B), suggest a role of tubular Bax and Bak in apoptosis in UUO.

### Double knockout of tubular Bax and Bak alleviates UUO-associated kidney fibrosis

**Figure 3:**
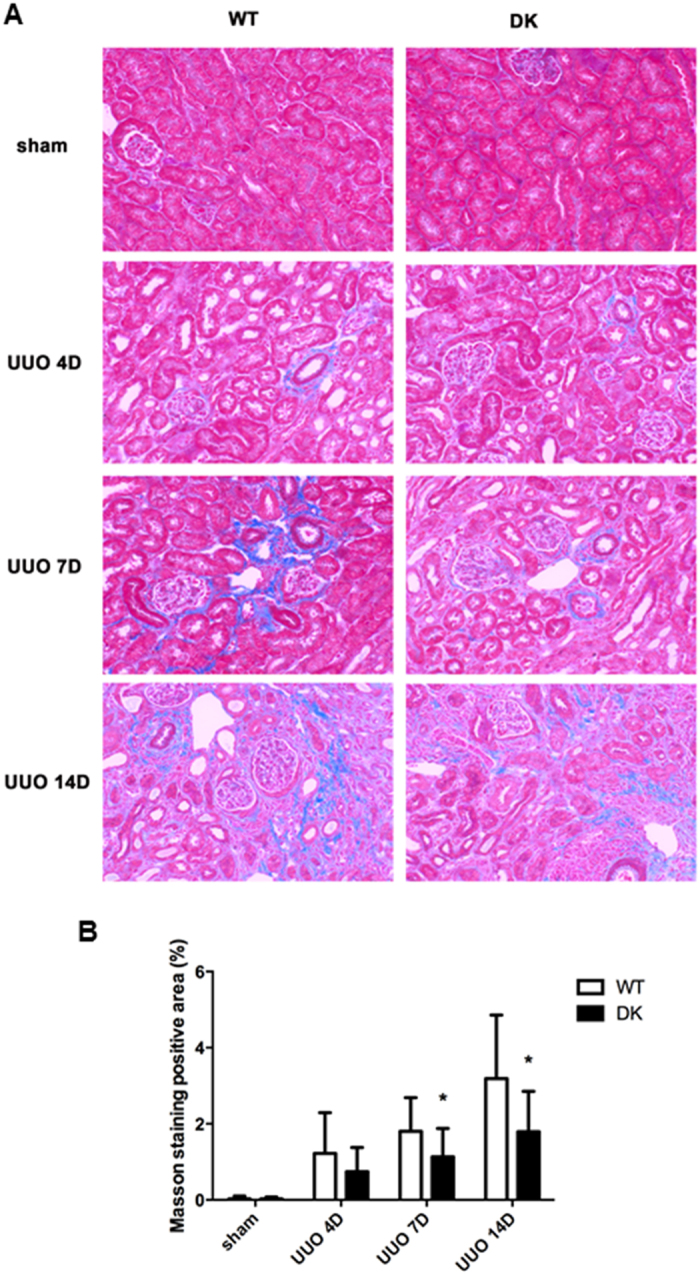
UUO-associated collagen deposition in kidney tissues is reduced in PT-Bax/Bak-DK mice. PT-Bax/Bak-DK and littermate WT mice (10–12 weeks old) were subjected to UUO or sham surgery, and then sacrificed to harvest the kidneys at 4, 7 and 14 days for paraffin-embedding and Masson’s trichrome staining. (**A**) Representative images of Masson’s trichrome staining. (**B**) Quantification of Masson’s trichrome positive areas in whole kidney tissue fields. Data were expressed as mean ± SD. *P < 0.05, significant difference between WT and PT-Bax/Bak-DK groups.

**Figure 4:**
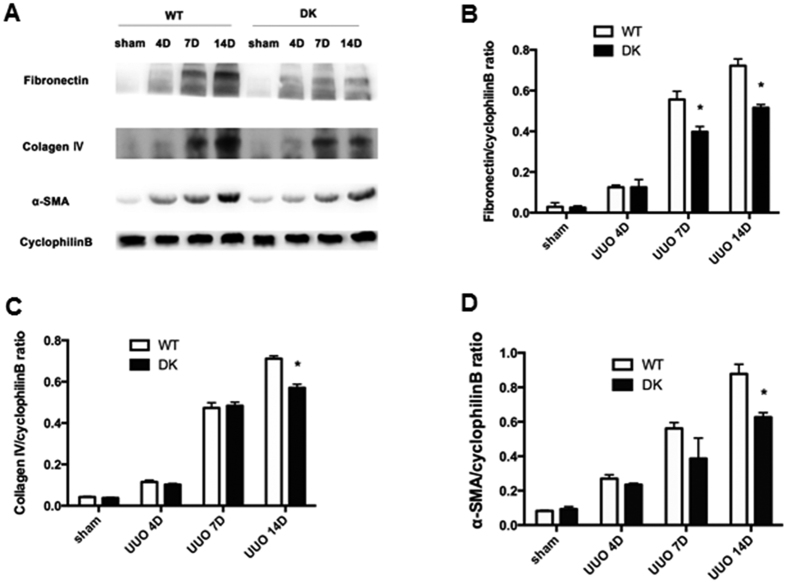
UUO-associated expression of fibrosis and extracellular matrix markers in kidney tissues is suppressed in PT-Bax/Bak-DK mice. PT-Bax/Bak-DK and littermate WT mice (10–12 weeks old) were subjected to UUO or sham surgery, and then sacrificed to harvest the kidneys at 4, 7 and 14 days. (**A**) Immunoblot analysis of kidney tissues for fibronectin, α-SMA, collagen IV and cyclophilin B (loading control). (**B**) Densitometry analysis of the immunoblot signals of fibronectin, α-SMA, and collagen IV as a ratio to cyclophilinB. Data were expressed as mean ± SD. *P < 0.05, significant difference between WT and PT-Bax/Bak-DK groups.

In order to elucidate the effect of Bax and Bak gene knockout in renal interstitial fibrosis, we detected collagen deposition by Masson’s trichrome staining and the expression of fibrotic hallmark proteins^9^,^10^. As shown by Fig. 3A, there was a time-dependent increase in blue Masson’s trichrome staining during UUO. Importantly, the wild type tissues clearly displayed higher Masson’s trichrome staining than PT-Bax/Bak-DK kidney tissues in renal interstitial area at 7 and 14 days of UUO, but no notable difference at 4 days of UUO (Fig. 3A and B). Consistently, the level of fibronectin, a fibrotic and ECM marker was markedly lower in PT-Bax/Bak-DK kidney tissues after 7 days and 14 days of UUO than wild type tissues (Fig. 4). The expression of another ECM protein Collagen IV was also lower in PT-Bax/Bak-DK kidney tissues at 14 days of UUO, although no difference was detected between the tissues at 7 days. Similarly, the expression ofα-SMA, a characteristic protein marker of myofibroblasts^11^, was also suppressed at 14 days of UUO in PT-Bax/Bak-DK mice (Fig. 4). These results demonstrate that ablation both Bax and Bak alleviates the deposition of ECM and fibrotic proteins, characteristics of renal interstitial fibrosis.

## Discussion

Apoptosis, as a form of programmed cell death, occurs during development and post-development pathogenesis. Especially when tissues or organs are under pathological situations, such as ischemia, hypoxia, toxicity, and metabolic stress, cells may undergo apoptosis^5^,^12^,^13^. A significant body of evidence has demonstrated two main pathways of apoptosis: the extrinsic and intrinsic pathways. Bax and Bak are two pro-apoptotic proteins of the Bcl-2 family that contain four BH (Bcl-2 homology) domains^14^,^15^,^16^. Bax normally resides in cell cytosol, whereas Bak is constitutively expressed on mitochondria. Upon cell stress, Bax is activated to translocate to mitochondria and cooperate with Bak to induce the permeabilization of mitochondrial outer membrane for the release of apoptogenic factors including cytochrome c to activate the intrinsic pathway of apoptosis. In the absence of Bax and Bak, stress-related apoptosis is diminished, supporting a critical role of Bax and Bak in the extrinsic pathway of apoptosis^17^,^18^. In kidneys, our previous work showed Bax activation in cisplatin-induced acute kidney injury and demonstrated that germline knockout of Bax could reduce cisplatin-induced renal apoptosis in mice^19^. Moreover, we demonstrated a critical role of Bax and Bak in ischemic AKI by using global and proximal tubule-specific knockout mouse model^5^. In that study, we noticed that global knockout of Bax did not protect againt ischemic AKI because of the enhanced neutrophil infiltration, but proximal tubule specific knockout of Bax could ameliorate ischemic AKI. In contrast, global Bak knockout mice model displayed protective effects from ischemic AKI by preserve mitochondrial dynamics and integrity^5^. It is noteworthy that Bax and Bak knockout mice attenuated apoptosis in ischemic AKI without affecting tubular necrosis^5^, suggesting a specific role of Bax and Bak in the regulation of apoptosis in kidneys. Despite these studies, the role of Bax, Bak and the intrinsic pathway of apoptosis in chronic conditions of kidney diseases remains less clear. In this study, we established a mouse model of double knockout of Bax and Bak from renal proximal tubules and used this model to demonstrate the role of the intrinsic pathway of apoptosis in UUO-associated apoptosis and interstitial fibrosis.

What role does tubular apoptosis play in fibrosis and renal pathologies in CKD or chronic kidney disease? Johnson and Dipietro showed that apoptosis had roles in the initiation and propagation of organ fibrosis via directly or indirectly pathways^20^. Indirectly, apoptosis may elicit inflammation to stimulate fibrosis. In this regard, upon activation macrophages may secrete profibrotic cytokines and growth factors, such as tumor necrosis factor α(TNFα), interleukin (IL)-6, IL-10 and transforming growth factor β1 (TGF-β1)^21^,^22^. On the other hand, apoptotic cells may directly affect the fibroblast cells and myofibroblasts to enhance their proliferation and activation into profibrotic phenotypes^3^,^23^,^24^. In our study, we detected apoptotic cells in wild-type kidney tissues mostly in the outer strip of out medulla beginning at UUO 4 day and increasing gradually after UUO 7 days and 14 days. Compared to wild-type, PT-Bax/Bak-DK tissues showed less apoptotic cells in each time point after UUO. Along with apoptosis, there was a continuous increase of renal fibrosis in wild-type tissues, which was also suppressed in PT-Bax/Bak-DK mice, indicating a correlation of tubular cell apoptosis and interstitial fibrosis (Fig. 3A and B). These results, together with a recent study^25^, support a role of tubular cell apoptosis in fibrogenesis in the intersitium. Of note, in our study there was residual apoptosis in PT-Bax/Bak-DK mice during UUO (Fig. 2). One possibility is that in addition to Bax/Bak-mediated mitochondrial pathway, other pathways of apoptosis, e.g. death receptor-mediated extrinsic pathway, may also contribute to tubular cell apoptosis in UUO. Alternatively, in the PT-Bax/Bak-DK model, Bax and Bak ablation was expected to occur in 70–80% proximal tubule cells due to the efficacy of PEPCK-Cre expression^26^, and the remaining 20–30% cells still contained Bax and Bak for apoptosis. Finally, some of the residual apoptosis might occur in other cell types such as distal tubules.

In conclusion, this study has established a renal proximal tubule Bax/Bak double knockout mouse model. By using this model, we have demonstrated a role of Bax/Bak-mediated intrinsic pathway in renal apoptosis during UUO. We further suggest that renal tubular apoptosis contributes to interstitial fibrosis.

## Materials and Methods

### Animals

The mouse line with floxed Bax gene was originally obtained from Dr. Stanley Korsmeyer at Dana-Farber Cancer Institute (Boston, MA)^27^. The mouse with floxed Bak gene were generated in our laboratory by a standard transgenic procedure. Both mouse lines have a mixed 129 and C57BL/6 background. PEPCK-CRE mouse line was kindly provided by Dr. Volker Haase at Vanderbilt University school of Medicine (Nashville, TN)^26^. These mice were crossed for several generations as depicted in Fig. 1A to produce renal proximal tubule-Bax/Bak double knockout mice (PT-Bax/Bak-DK) and wild type mice (WT). The genotypes of the mice were verified by PCR-based genotyping. Male animals aged 10–12 weeks were used in this study. All animals were maintained at Charlie Norwood VA Medical Center under 12-hour light/12-hour dark pattern with free access to food and water. All experiments were carried out according to the protocol approved by the Institute Animal Care and Usage Committee in Charlie Norwood VA Medical Center, Augusta, GA.

### Regents

The sources of primary antibodies were as follows: Fibronectin, α-SMA, Collagen Ι and Collagen IV were purchased from Abcam (Cambridge, MA), and β-actin was from Sigma (St Louis, MO). All second antibodies were purchased from Jackson Immuno Research Laboratories Inc (West Grove, PA). Paraformaldehyde was from Sigma (St. Louis, MO).

### Animal surgery

PT-Bax/Bak-DK and WT mice were anesthetized by intraperitoneal injection with 60 mg/kg pentobarbital. To relieve distress, 0.05 mg/kg buprenex was given after surgery. During surgery, mice were kept on a temperature-controlled operating table to maintain body temperature. The left ureter was dissected out and ligated by 4.0 silk at two points along the length, while the sham group underwent the same procedure except for ureter ligation. After 4, 7, 14 days of UUO, mice were sacrificed and kidney were harvested for pathological and immunoblotting analyses.

### Immunoblot analysis

Kidney tissue lysates were extracted with 2% SDS buffer as previously^28^. Protein amounts were determined with the BCA kit from Thermo Scientific and equal amounts of protein were loaded in each lanes for SDS-polyacrylamide electrophoresis and then transferred to PVDF membrane. The blot was blocked with 5% milk for 1 hour at room temperature and then incubated with a specific primary antibody. The blot membrane was washed with TBST for 4 times and then incubated with horseradish peroxidase-conjugated secondary antibody, and signals were revealed using a chemiluminescence kit (Thermo Scientific).

### Masson’s trichrome staining of fibrosis

Renal fibrosis was shown by Masson’s trichrome staining according to a standard protocol as described recently^29^. Briefly, the tissue was stained with hematoxylin, and then with ponceau red liquid dye acid complex, followed by incubation with phosphomolybdic acid solution. Finally, the tissue was directly stained with aniline blue liquid and acetic acid. The percentage of Masson staining positive area was determined in 10–20 microscopic fields for each sample, and ImageJ was used for quantitatively evaluation.

### TUNEL assay

TUNEL assay was conducted by using the *in situ* Cell Death Detection kit from Roche as previously^29^,^30^. Paraffin-embedded tissue were deparaffinized and permeabilized with 0.1 M sodium citrate, pH 6.0 at 65 °C for 1 hour. Then, the tissue section was incubated with the TUNEL reaction buffer for 1 hour at 37 °C in a humidified chamber. The slides were mounted with ProLong Antifade Reagent. Positive staining was detected by Axioplan2 fluorescence microscope. In each tissue section, 10–20 fields were selected randomly to count positive staining cells.

### Statiscal analysis

All values were expressed as mean ± SD. Comparison between two groups was performed by paired Student t-test or unpaired student t-test. ANOVA were used to compare multiple groups. Data were analyzed with a Prism 6.0 software packet (San Diego, CA). Significance was considered by a *P* value of <0.05.

## Additional Information

**How to cite this article:** Mei, S. *et al*. Double knockout of Bax and Bak from kidney proximal tubules reduces unilateral urethral obstruction associated apoptosis and renal interstitial fibrosis. *Sci. Rep.* **7**, 44892; doi: 10.1038/srep44892 (2017).

**Publisher's note:** Springer Nature remains neutral with regard to jurisdictional claims in published maps and institutional affiliations.
